# Examining rurality and social determinants of health among women with GDM: a 15-year comprehensive population analysis

**DOI:** 10.1186/s12905-024-03306-6

**Published:** 2024-08-24

**Authors:** Umama Ali, Laila Cure, Rhonda K. Lewis, Ajita Rattani, Twyla Hill, Nikki Keene Woods

**Affiliations:** 1 Department of Public Health Sciences, Wichita State University, Wichita, KS USA; 2 Industrial, Systems, and Manufacturing Engineering Department, Wichita State University, Wichita, KS USA; 3 Department of Psychology, Wichita State University, Wichita, KS USA; 4 Department of Electrical Engineering and Computer, Wichita State University, Wichita, KS USA; 5 Department of Sociology, Wichita State University, Wichita, KS USA

**Keywords:** Gestational diabetes mellitus, Maternal obesity, Rural, Rural-urban, Obese pre-pregnancy BMI, Rural health, Health disparities, Pregnancy, Women’s health

## Abstract

**Background:**

Gestational diabetes mellitus (GDM) is a common pregnancy complication with long-term health consequences for mothers and their children. The escalating trends of GDM coupled with the growing prevalence of maternal obesity, a significant GDM risk factor projected to approach nearly 60% by 2030 in Kansas, has emerged as a pressing public health issue.

**Methods:**

The aim of this study was to compare GDM and maternal obesity trends in rural and urban areas and investigate maternal demographic characteristics influencing the risk of GDM development over a 15-year period. Trend analyses and a binary logistic regression were employed utilizing 2005 to 2019 de-identified birth record vital statistics from the Kansas Department of Health and Environment (*N* = 589,605).

**Results:**

Over the cumulative 15-year period, a higher prevalence of GDM was observed across age, race/ethnicity, education, and insurance source. Throughout this period, there was an increasing trend in both GDM and obese pre-pregnancy BMI age-adjusted prevalence, with noticeable rural-urban disparities. From 2005 to 2019, women, including Asians (OR: 2.73, 95% CI 2.58%-2.88%), American Indian or Alaskan Natives (OR: 1.58, 95%, CI 1.44-1.73%), Hispanics (OR: 1.42, 95% CI 1.37%-1.48%), women residing in rural areas (OR: 1.09, 95%, CI 1.06-1.12%), with advanced maternal age (35–39 years, OR: 4.83 95% CI 4.47%-5.22%; ≥40 years, OR: 6.36 95%, CI 5.80-6.98%), with lower educational status (less than high school, OR: 1.15, 95% CI 1.10%-1.20%; high school graduate, OR: 1.10, 95% CI 1.06%-1.13%), Medicaid users (OR: 1.10, 95% CI 1.06%-1.13%), or with an overweight (OR: 1.78, 95% CI 1.72%-1.84%) or obese (OR: 3.61, 95% CI 3.50%-3.72%) pre-pregnancy BMI were found to be at an increased risk of developing GDM.

**Conclusions:**

There are persistent rural-urban and racial/ethnic disparities present from 2005 to 2019 among pregnant women in Kansas with or at-risk of GDM. There are several socioeconomic factors that contribute to these health disparities affecting GDM development. These findings, alongside with prominent rising maternal obesity trends, highlight the need to expand GDM services in a predominantly rural state, and implement culturally-responsive interventions for at-risk women.

## Introduction

Gestational diabetes mellitus (GDM) is a form of diabetes that occurs exclusively during pregnancy, developed in the second and third trimester of pregnancy. In the last three decades, there has been a growing prevalence of GDM, currently affecting approximately 7% of all pregnancies, although the prevalence may range from 1 to 14% depending on the population studied and the diagnostic tests applied [1, 2]. GDM increases the likelihood for adverse maternal and infant health outcomes [3, 4]. Women with GDM face a significantly higher probability of developing type 2 diabetes, with the risk being at least seven-times greater or about 50% among those with GDM, making it a strong predictor of future type 2 diabetes [2, 5]. Additionally, women with GDM may also face an elevated risk for hypertension, maternal morbidity and mortality, spontaneous preterm birth, cesarean delivery, and preeclampsia [4–6]. The impact of GDM extends beyond pregnancy as infants born to women with GDM are at an increased likelihood of macrosomia, neonatal respiratory distress syndrome, increased perinatal mortality, long-term childhood obesity, and diabetes in late adolescence and young adulthood [4, 6].

Well-documented risk factors for GDM include advanced maternal age, family history of diabetes, nonwhite race/ethnicity, overweight and obese body mass index (BMI) or maternal weight, lack of physical activity, history of GDM, parity, cigarette smoking and low socioeconomic status (SES) such as low income [1, 6–9]. Maternal obesity is a significant and strongly associated risk factor for GDM suggesting that nearly 50% of GDM cases could be prevented if women had a healthy BMI [3, 8–10]. The prevalence of pre-pregnancy obesity has increased since 2019 across all maternal ages, race and Hispanic-origin ethnicities, and educational levels [11, 12].

Significant health disparities exist between rural and urban women. Rural women are at increased odds of having an overweight or obese pre-pregnancy BMI and experience higher rates of GDM [13]. Previous research have shown that rural women had a higher frequency of GDM compared to urban women, with a statistically significant adjusted risk ratio of 1.17 observed from 2011 to 2019 [13]. Rural areas face additional challenges including environmental risk factors such as limited resources for diabetes self-management, inaccessibility to maternity services and quality-care, scarcity of healthcare providers, inadequate public transportation, and frequent obstetric unit closures in low-income communities. These issues are compounded by individual risk factors such as unhealthy diets, higher obesity rates, lower educational attainment, and being a Black, non-Hispanic women, factors all linked to poorer health outcomes [14–18]. These disparities may contribute to poor prevention and management of GDM in rural areas.

As of 2020, the national GDM rate was 7.8% in the United States compared to Kansas with a higher rate of 8-8.9% [19]. As reported by the CDC in 2022, the current obesity rate in Kansas was 35.7%. Current projections in a prior study indicate that obesity prevalence in Kansas, especially among women, is expected to reach nearly 60% by 2030 [20, 21]. In this majority rural state, these growing public health concerns are compounded with 45.7% of counties defined as maternity care deserts compared to national 32.6% in the US [22]. There is a critical need to investigate GDM trends and associated potential risk factors linked to rurality and maternal demographic traits, aspects that remain unexplored in Kansas. Our study focuses on a population-level, comprehensive analysis of GDM over the 15-year period from 2005 to 2019. We analyze the estimated GDM and maternal obesity age-adjusted prevalence stratified by rurality through trend analyses, while employing a logistic regression model to investigate contributing factors of women at risk for GDM development in a predominantly rural state.

## Methods

### Sample

A retrospective secondary analysis was performed to analyze GDM trends and employ a logistic regression model to assess GDM probability in women who gave live births in Kansas, using 2005–2019 birth vital statistics records from the Kansas Department of Health and Environment (KDHE). Birth vital statistics are compiled from birth certificates, filed by the state law with the Bureau of Epidemiology and Public Health Informatics at KDHE. Institutional Review Board approval was acquired prior to the study and the data were de-identified.

### Analysis

All analyses were limited to women with live births (*n* = 589,605). Descriptive statistics were used to summarize key maternal demographics and health status of the participants, including age, race, Hispanic origin, educational level, insurance source, employment status, and pre-pregnancy BMI (Table 1).

The GDM and pre-pregnancy BMI data collected over 15-years among women with confirmed GDM or with pre-pregnancy overweight or obesity were analyzed to identify longitudinal trends. The prevalence for each variable was aggregated annually. Furthermore, the interplay of rurality with GDM and pre-pregnancy BMI was analyzed by stratifying women into rural and urban groups. All prevalence rates were age-standardized to the female U.S. population in 2000, as reported by the U.S. Census Bureau, with the following age-groups: <20 (12–19 years), 20–29, 30–39, and ≥ 40 (40–69 years).

### Variables

Rurality was defined and categorized by KDHE in which Kansas counties were grouped into five geographical peer groups according to the average number of persons per square mile (ppsm): frontier (less than 6.0 ppsm), rural (6.0-19.9 ppsm), densely settled rural (20.0-39.9 ppsm), semi-urban (40-149.9 ppsm), and urban (150.0 or more ppsm). For this study, semi-urban and urban peer groups were combined into one urban group while frontier, rural, and densely settled rural were combined into one rural group, for simplicity.

GDM, as defined by the KDHE, refers to any level of glucose/carbohydrate intolerance that occurs or is first identified during pregnancy, based on the diagnostic and classification criteria established by the American Diabetes Association and listed on birth certificates [23]. Pre-pregnancy BMI was calculated by KDHE using the height and pre-pregnancy weight of the birth mother using the standard BMI calculation method. It was further categorized based on the World Health Organization’s BMI classification: underweight (BMI < 18.5 kg/m2), healthy (BMI is 18.5–24.9 kg/m2), overweight (BMI is 25–29.9 kg/m2) and obese (BMI ≥ 30 kg/m2).

Demographic characteristics included age (< 20, 20–24, 25–29, 30–34, and 35–39, ≥ 40), race (White, Black, American Indian or Alaskan Native, Asian, and other multiracial; not including Hispanic origin), Hispanic origin defined as if the mother was Spanish, Hispanic or Latina, educational attainment (some college and college graduate, less than high school, high school graduate, more than college, and unknown), insurance source (Medicaid, Private and other (self-pay, Indian health service, CHAMPUS/TRICARE, other government, other and unknown)), and employment (employed, unemployed, or unknown/unspecified/unwilling to divulge).

### Statistical modeling

All statistical analyses were performed on IBM SPSS Software version 28. The demographics and health status categorical variables were expressed as frequencies and percentages, and compared using the Pearson chi-square test. A binary logistic regression was performed to investigate the potential of women developing GDM according to various demographic characteristics. Women were classified into one of two groups based on the dichotomous dependent outcome variable, have GDM or don’t have GDM. The dependent variable was coded as a binary category with no GDM being represented as 0 and GDM as 1. Predictor variables considered in the model as categorical independent variables included age, race, Hispanic origin, educational status, insurance source, employment, pre-pregnancy BMI, and rurality. Reference groups for each predictor variable was assigned on those with low risk for GDM according to prior literature, defined as women < 20 years of age, white, is Hispanic, has some college education or college graduate, utilize private insurance, is employed, has a pre-pregnancy BMI in the healthy weight range, and lives in urban areas. A collinearity test was performed prior to the analysis to check for correlations between the independent variables. Records with missing variables were excluded from the logistic regression analysis. From the 589,605 live births in this dataset, 551,053 were included in the logistic regression analysis (38,552 or 6.54% had missing variables and were excluded from the analysis).

## Results

### Sample demographic characteristics

Among women who gave live birth from 2005 to 2019 (*n* = 589,605) the majority were between the ages of 20 and 34 (80.1%), white (81.8%), not Hispanic (83.7%), had some college or college graduate educational level (51.3%), utilized private insurance (52.6%), were employed (92.7%), and had a healthy pre-pregnancy BMI (44.7%; Table 1).

**Table 1:** Kansas Women’s Live Birth Demographics (2005–2019): Rural vs. Urban GDM Prevalence

Characteristic	Overall	Women with GDM		Urban Women with GDM**		Rural Women with GDM**	
	N (%)*	N (%)	*p*-value	N (%)	*p*-value	N (%)	*p*-value
**Total**	**589,605**	29,448 (5.0)		18,864 (4.9)	< 0.001	10,583 (5.2)	< 0.001
**Age group (yrs)** ^☉^					< 0.001		< 0.001
< 20	48,105 (8.2)	900 (1.9)	< 0.001	516 (1.8)		384 (2.0)	
20–24	148,893 (25.3)	4,462 (3.0)		2,493 (2.8)		1,969 (3.2)	
25–29	182,691 (31)	8,394 (4.6)		5,224 (4.4)		3,170 (4.9)	
30–34	140,384 (23.8)	9,141 (6.5)		6,192 (6.2)		2,948 (7.4)	
35–39	57,680 (9.8)	5,202 (9.0)		3,517 (8.4)		1,685 (10.7)	
≥ 40	11,640 (2.0)	1,349 (11.6)		922 (11.1)		427 (13.0)	
**Race**			< 0.001		< 0.001		< 0.001
White	482,184 (81.8)	23,177 (4.8)		13,973 (4.7)		9,203 (5.1)	
Black	45,376 (7.7)	1,708 (3.8)		1,467 (3.7)		241 (4.0)	
Asian	18,885 (3.2)	1,838 (9.7)		1,638 (9.9)		200 (8.4)	
American Indian or Alaskan Native	7,869 (1.3)	583 (7.4)		321 (7.3)		262 (7.6)	
Other	35,061 (5.9)	2,139 (6.1)		1,462 (5.9)		677 (6.5)	
**Hispanic Origin** ^**§**^			< 0.001		< 0.001		< 0.001
Yes	95,719 (16.2)	6,241 (6.5)		3,756 (6.3)		2,485 (6.8)	
No	493,449 (83.7)	23,207 (4.7)		15,108 (4.6)		8,098 (4.8)	
**Education**			< 0.001		0.007		0.002
Less than high school	89,575 (15.2)	4,620 (5.2)		2,686 (4.9)		1,934 (5.5)	
High school graduate	138,986 (23.6)	6,698 (4.8)		4,100 (4.7)		2,597 (4.9)	
Some college	129,413 (22.0)	6,598 (5.1)		4,062 (5.1)		2,536 (5.1)	
College graduate	172,443 (29.3)	8,532 (4.9)		5,688 (4.8)		2,844 (5.2)	
More than college	55,156 (9.4)	2,856 (5.2)		2,243 (5.1)		613 (5.6)	
Unknown	2,271 (0.4)	134 (5.9)		79 (5.8)		55 (6.1)	
**Insurance source for delivery**			< 0.001		< 0.001		< 0.001
Medicaid	176,201 (29.9)	8,607 (4.9)		5,095 (4.8)		3,511 (5.0)	
Private	310,195 (52.6)	16,267 (5.2)		10,862 (5.1)		5,405 (5.5)	
Other¶	98,767 (16.8)	4,553 (4.6)		2,895 (4.5)		1,658 (4.8)	
**Employment**			0.244		0.215		0.017
Employed	546,505 (92.7)	27,578 (5.0)		17,863 (4.9)		9,715 (5.3)	
Unemployed	11,768 (2.0)	554 (4.7)		308 (5.0)		246 (4.4)	
Unknown/unspecified/unwilling to divulge	2,353 (0.4)	116 (4.9)		37 (3.7)		79 (5.8)	
Pre-pregnancy BMI***			< 0.001		< 0.001		< 0.001
Underweight	22,331 (3.8)	452 (2.0)		317 (2.1)		135 (1.9)	
Healthy	263,716 (44.7)	7289 (2.8)		4,958 (2.8)		2,331 (2.7)	
Overweight	149,000 (25.3)	7,421 (5.0)		4,852 (5.1)		2,569 (4.8)	
Obese	143,205 (24.3)	13,716 (9.6)		8,362 (9.5)		5,354 (9.7)	

*The number of women in a characteristic group (e.g., age group, race) may not sum to the total number of women due to missing information in the corresponding variable

**Peer Group is defined by Kansas Department of Health and Education (KDHE) categorizing counties in Kansas into five groups based on person per square mile (ppsm): urban (≥ 150.0 ppsm), semi-urban (40.0-149.9 ppsm), densely settled rural (20.0-39.9 ppsm), rural (6.0-19.9 ppsm), frontier (< 6.0 ppsm). Urban peer group combines urban and semi-urban groups. Rural peer group combines densely settled rural, rural and frontier groups. The reported percentages are women with only a positive GDM diagnosis per each peer group. Women residing in urban and rural areas are then further categorized with a GDM diagnosis were reported

^☉^ Women aged 12–19 were classified as being under the age of 20 while those aged 40–69 were categorized as 40 years old and older

§ Hispanic origin was reported separately from race. Women that reported Hispanic origin were categorized as Hispanic regardless of race

¶Includes self-pay, other insurances provided by Indian Health Service, CHAMPUS/TRICARE or other government, other and unknown payment methods

*** Pre-pregnancy body mass index (BMI) was classified according to World Health Organization as underweight (BMI > 18.5), healthy weight (BMI 18.5–24.9), overweight (BMI 25-29.9) and obese (BMI < 30)

Analysis of the dataset showed that 5% of births were complicated by GDM (Table 1). Women with GDM were significantly older (11.6% at ≥ 40 years vs. 3% with 20–24 years), Asian (9.7%) and American Indian or Alaskan Native (7.4%), Hispanics (6.5%), utilized private insurance (5.2%), and had an overweight (5%) or obese pre-pregnancy BMI (9.6%) compared to women without GDM (all *p* < 0.001). GDM prevalence was similar across all levels of educational attainment.

There was a higher GDM prevalence among women residing in rural areas (5.2%) than their urban counter parts (4.9%; *p* < 0.001). Even among women residing in urban and rural areas, these prevalence trends are similarly demonstrated. Age was a statistically significant predictor of GDM prevalence, with older women having a statistically higher GDM prevalence (*p* < 0.001). Across both rurality groups, racial disparities were evident as Asian (9.9% and 8.4%) and American Indian or Alaskan Native (7.3% and 7.6%) women residing in either urban or rural areas, respectively, had a higher prevalence compared to White women. Hispanic origin showed higher GDM prevalence in urban (6.3%) and rural (6.8%) settings (*p* < 0.001). Privately insured women in both urban and rural areas had higher prevalence rates compared to those covered by Medicaid or other insurance types (*p* < 0.001). Among rural women, employment status was marginally associated with GDM (*p* = 0.017), with unemployed women showing a lower prevalence (4.4%) compared to employed women (5.3%). Pre-pregnancy BMI remained a significant predictor of GDM in both urban (9.5%) and rural (9.7%) populations (*p* < 0.001), with women having a pre-pregnancy obese BMI demonstrating the highest prevalence.

### Trends of GDM and maternal obesity

**Fig. 1:**
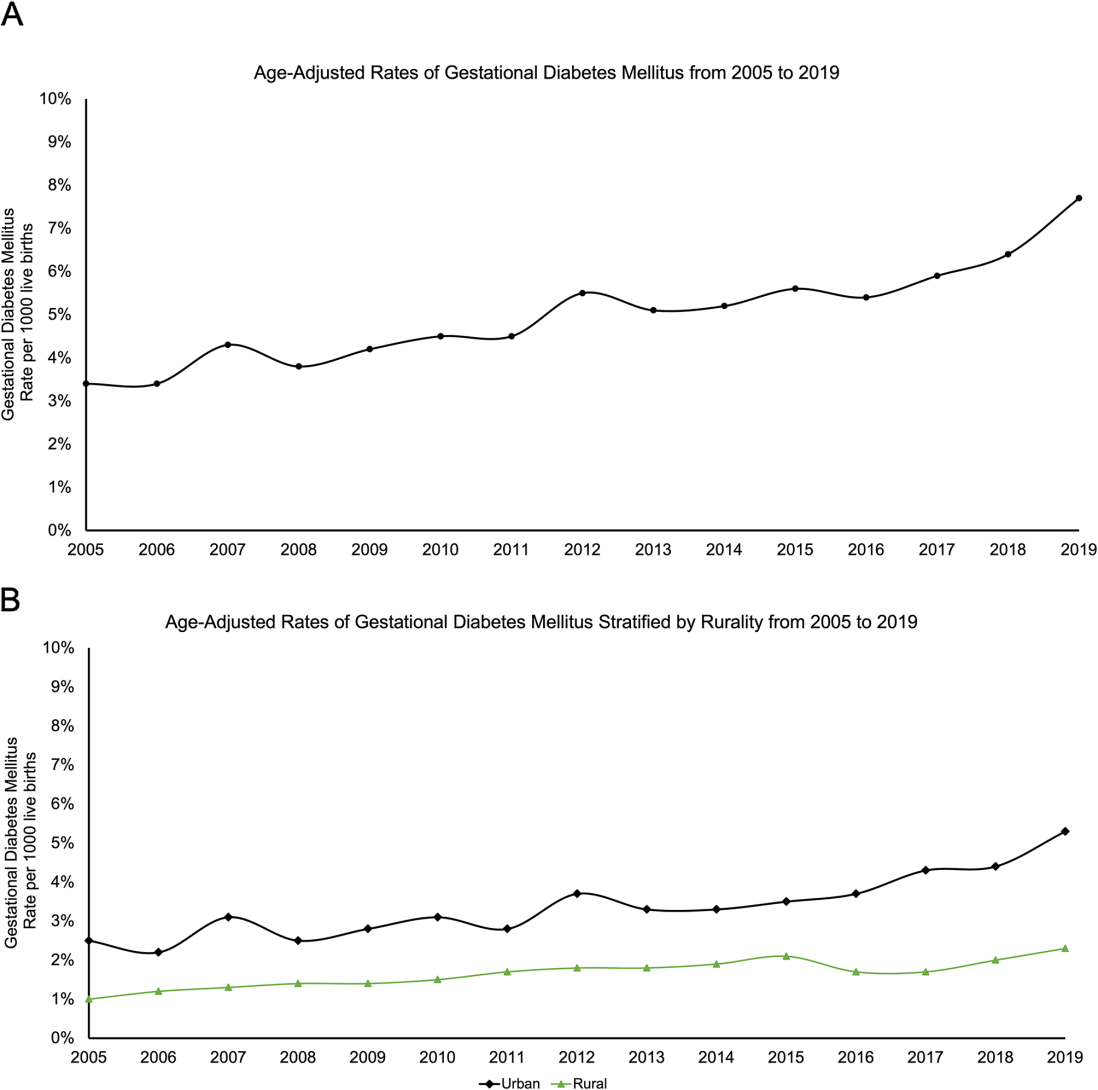
**A.** Age-adjusted annual prevalence of all women who gave live births with gestational diabetes mellitus in Kansas from 2005–2019. **B.** Age-adjusted annual prevalence of all women who gave live births, stratified by rurality into urban and rural groups, with gestational diabetes mellitus in Kansas from 2005–2019

**Fig. 2:**
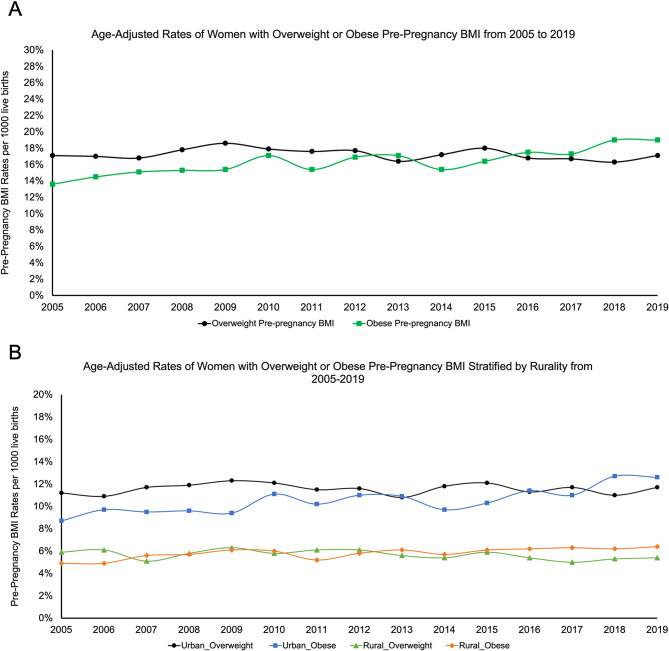
**A.** Age-adjusted annual prevalence of all women who gave live births with overweight or obese pre-pregnancy BMI in Kansas from 2005–2019. **B.** Age-adjusted annual prevalence of all women who gave live births, stratified by rurality into urban and rural groups, with overweight or obese pre-pregnancy BMI in Kansas from 2005–2019

Over the span of 15 years, the age-adjusted prevalence of GDM per 1,000 live births rose significantly from 3.4% in 2005 to 7.7% in 2019, averaging 5% (Fig. 1A). Furthermore, urban women saw an increase in GDM prevalence from 2.5 to 5.3% over the same period, while rural women experienced a rise from 1 to 2.3% (Fig. 1B). Among women with overweight or obese pre-pregnancy BMI, significant trends in maternal obesity were observed. The age-adjusted prevalence per 1,000 live births among those with an overweight BMI showed a general stability over the 15-years (Fig. 2A). Conversely, for women with an obese pre-pregnancy BMI, the prevalence increased from 13.6% in 2005 to 19.0% in 2019. Analyzing rural-urban disparities, both urban and rural women with an overweight pre-pregnancy BMI exhibited consistent trends across the 15-years. In contrast, urban women with an obese pre-pregnancy BMI saw an increase from 8.7% in 2005 to 12.6% in 2019, while their rural counterparts experienced a rise from 4.9% in 2005 to 6.4% in 2019 (Fig. 2B).

### Odds of developing GDM

None of the predictor variables in the binary logistic regression had multicollinearity as indicated by tolerance (> 0.1) and variance inflation factor (< 2) values. Women with an overweight or obese pre-pregnancy BMI were found to be at a significantly higher risk, being 1.78 (95% CI 1.72%-1.84%, *p* < 0.001) and 3.61 (95% CI 3.5%-3.72%, *p* < 0.001) times more likely, respectively, to develop GDM compared to those with a healthy pre-pregnancy BMI (Table 2). Interestingly, women residing in rural areas showed only a modest 9% increase (OR: 1.09, 95% CI 1.06%-1.12%, *p* < 0.001) in the likelihood of GDM compared to their urban counterparts. For racial backgrounds, American Indian or Alaskan Native and Asian women had substantially higher odds of GDM, with 1.58- (95% CI 1.44%-1.73%, *p* < 0.001) and 2.73-times (95% CI 2.58%-2.88%, *p* < 0.001) more likely, respectively, when compared to White women. Additionally, Hispanic women demonstrated a 42% elevated (OR: 1.42, 95% CI 1.37%-1.48%, *p* < 0.001) likelihood of GDM relative to non-Hispanic women. Older women, particularly those 40 and above, demonstrated an elevated likelihood of GDM, with a risk approximately 6.36 times (95% CI 5.80%-6.98%, *p* < 0.001) higher than that of women younger than 20 years old. Women with higher levels of education such as those with more than college level (OR: 0.92, 95% CI 0.88%-0.96%, *p* < 0.001) and those utilizing private insurance displayed lower GDM risk in contrast to women with lower educational attainment or Medicaid insurance utilization (OR: 1.10, 95% CI 1.06%-1.13%, *p* < 0.001). Lastly, employment status was not identified as a significant predictor in our analysis (Table 2).

**Table 2:** GDM parameter estimates with pre-pregnancy BMI and demographic variables

Variable	OR [95% CI]	*p* value
**Pre-pregnancy BMI**		
Healthy	REF	
Underweight	0.79 [0.71, 0.87]	< 0.001
Overweight	1.78 [1.72, 1.84]	< 0.001
Obese	3.61 [3.50, 3.72]	< 0.001
**Peer Groups**		
Urban	REF	
Rural	1.09 [1.06, 1.12]	< 0.001
**Race**		
White	REF	
Black	0.80 [0.76, 0.85]	< 0.001
American Indian or Alaskan Native	1.58 [1.44, 1.73]	< 0.001
Asian	2.73 [2.58, 2.88]	< 0.001
Other	0.96 [0.91, 1.02]	0.169
**Hispanic Origin**		
Not Hispanic	REF	
Hispanic	1.42 [1.37, 1.48]	< 0.001
**Age**		
< 20	REF	
20–24	1.49 [1.38, 1.60]	< 0.001
25–29	2.40 [2.23, 2.58]	< 0.001
30–34	3.57 [3.31, 3.85]	< 0.001
35–39	4.83 [4.47, 5.22]	< 0.001
≥ 40	6.36 [5.80, 6.98]	< 0.001
**Education**		
Some college and college graduate	REF	
Less than high school	1.15 [1.10, 1.20]	< 0.001
High school graduate	1.10 [1.06, 1.13]	< 0.001
More than college	0.92 [0.88, 0.96]	< 0.001
Unknown	1.11 [0.89, 1.37]	0.364
**Insurance Source**		
Private	REF	
Medicaid	1.10 [1.06, 1.13]	< 0.001
Other	0.91 [0.87, 0.94]	< 0.001
**Employment**		
Employed	REF	
Unemployed	1.05 [0.96, 1.15]	0.246
Unknown/unspecified/unwilling to divulge	1.00 [0.82, 1.22]	0.975
Constant	0.01	< 0.001

## Discussion

### Main findings

The study provided insightful trends and disparities regarding GDM and maternal obesity between 2005 and 2019. The results demonstrate a significant increase in GDM cases, aligning with the upward trend in maternal obesity rates. Although urban women exhibited higher GDM prevalence compared to rural women, there was a gradual rise in GDM rates among rural women across the 15-years. Additionally, the study highlighted demographic disparities, indicating elevated risks among older women, Asian and American Indian or Alaskan Native populations, Hispanics, and individuals with overweight or obese pre-pregnancy BMIs.

### Interpretation

The overall increasing prevalence of GDM over a 15-year period aligns with findings from various prior studies, which have consistently reported rising GDM rates since 1989, utilizing diverse data sources and reporting methods [9, 19, 24–27]. According to CDC data, GDM prevalence surged by 56% from 2000 to 2010 and by an additional 30% from 2006 to 2019 [19, 27]. As of 2020, Kansas reported a GDM prevalence of 8.7% [19]. Simultaneously, the prevalence of maternal obesity has seen a significant increase, mirroring earlier literature and the broader adult obesity trend, which, in 2022, stood at 36%, with severe obesity (BMI ≥ 35) predicted to become the most common BMI category among women [1, 11, 12, 17, 21, 28]. In 2020, Kansas exhibited a 34% obesity rate among women, showcasing a higher-than-average trend [20, 28]. The parallel rise in maternal obesity and GDM rates highlight a growing concern, as these trends are expected to continue increasing.

This study sheds light on the rural-urban disparities among pregnant women with GDM from 2005 to 2019. The observed steady increase in the age-adjusted prevalence of GDM cases in the sample population and a slightly elevated risk of GDM among women residing in rural areas align with previous findings specific to GDM in rural areas within the United States [13]. However, there are very few studies comparing GDM prevalence based on rurality in the US. The trend analysis of GDM rates in rural versus urban areas consistently shows higher rates in rural areas at most time points, with anticipated increases over time. These geographic disparities further highlight the elevated risk of adverse pregnancy outcomes for women in rural areas which has health implications for both the mother and child [14–16, 18, 29]. Notably, one adverse outcome of GDM is the increased likelihood of developing type 2 diabetes [5]. These findings contribute to the broader concerns about health disparities, health equity, barriers to care, and the challenges of maternal care deserts in the state that affect factors such as access to quality care, and effective GDM prevention, screening, and management, including appropriate nutrition, which persist in rural areas [15, 16, 18, 30]. Recent data from Kansas reveals that nearly 20% of infants are born to women residing in rural counties, many of which are classified as maternity care deserts [22]. Concerning travel distances in Kansas, women on average, travel 10 miles to the nearest birthing hospital, while some may need to travel nearly 63 miles in counties with the longest travel times [22]. Moreover, women in 45% of Kansas counties face a very high or high vulnerability to adverse pregnancy outcomes, including increased risks of maternal morbidity [22].

These findings highlight health disparities among different demographic groups. Asians, American Indians or Alaskan Natives, and Hispanics exhibited the highest risks of GDM, aligning with prior research on racial and ethnic disparities in GDM rates. American Indians or Alaskan Natives showed particularly elevated rates of GDM, pre-pregnancy diabetes, and overweight or obesity, alongside greater likelihoods of inadequate prenatal care and disparities linked to social determinants of health [31]. Hispanics also experienced predictively higher GDM rates compared to non-Hispanic Whites [25, 27, 32]. Conversely, Black pregnant women exhibited a lower risk of GDM but faced heightened risks of developing type 2 diabetes compared to other racial and ethnic groups [10, 19, 27, 35]. Additionally, our findings reaffirm that Asians have the highest prevalence of GDM and are at heightened risk compared to other racial and ethnic groups, with CDC data from 2020 indicating non-Hispanic Asians had the highest GDM rate among the largest racial and Hispanic-origin groups, at 14.9% [19, 33]. An important consideration is the variability in BMI classifications across ethnicities. For instance, varying gestational weight gain guidelines for Chinese women, advocating 7–11 kg for those of normal weight and 6–8 kg for overweight or obese pregnant women, offer additional perspective on identifying women susceptible to GDM [36]. Future research should aim to investigate and validate BMI standards tailored to diverse racial and ethnic backgrounds, ensuring those at-risk are identified for targeted interventions, effectively mitigating the risk of GDM.

Our findings confirm the link between advanced maternal age and a significantly increased risk of GDM, consistent with previous research. According to CDC data from 2020, GDM prevalence rose steadily with maternal age, ranging from 2.5% among women under 20 to 15.3% among those aged 40 and older [19]. Previous studies further support this trend, demonstrating a linear increase in GDM risk across age groups, with odds ratios ranging from 0.60 (95% CI, 0.50–0.72) for women under 20 to 4.86 (95% CI, 3.78–6.24) for women aged 40 and older [34]. This association firmly establishes maternal age as a robust determinant of GDM risk [1, 19, 34, 37]. While the exact biological mechanisms underlying why older women are more susceptible to GDM remain uncertain, potential factors include heightened insulin resistance, elevated inflammatory markers and oxidative stress, and age-related impairments in carbohydrate metabolism [34, 37].

This study initiates an exploration into the connection between SES and GDM risk. The association between maternal education level and GDM development, particularly in the US, requires further investigation to establish a clear connection. One study demonstrated no significant association between maternal education level and GDM development [11]. Similar gaps exist for employment, with limited prior research on the precise correlation between employment and GDM. The literature concerning GDM has not comprehensively considered education and employment. Previous research consistently links SES to GDM. Lower SES, such as incomes below the poverty threshold (< 100%), is associated with a higher increase in GDM prevalence (4.3%) compared to higher income brackets [9]. Studies also show independent associations between low relative SES (OR: 2.04, 95% CI 1.07%-3.89%) and poverty (OR: 1.81, 95% CI 0.97%-3.38%) with GDM using logistic regressions [38]. Other studies demonstrate higher SES is inversely correlated with GDM (adjusted relative risk 0.710, 95% CI 0.507–0.995%) utilizing a bivariate probit model, indicating a socioeconomic gradient in GDM prevalence, with estimates suggesting an 8.6% increase (95% CI 2.7%-12.0%) in the lowest socioeconomic group compared to the highest [39]. Therefore, further exploration into the impact of socioeconomic status on GDM, encompassing education and employment, is essential, given that SES consistently emerges as a robust predictor of disease onset and progression [40]. Unexamined variables, such as income, may mediate the relationship between education and employment on GDM. Income, while not directly assessed, can be approximated using insurance source, revealing a higher GDM risk among Medicaid users, a crucial source of funding for nearly half of US births, particularly in rural areas [18]. Low-income pregnant women with Medicaid encounter challenges in accessing consistent and timely care, receiving less prenatal care, having fewer deliveries, and exhibiting a higher likelihood of obesity and smoking [18, 30]. Pregnant women covered by Medicaid also face elevated rates of severe maternal morbidity and mortality, emphasizing the need for targeted interventions and access to quality care [18]. Approximately 36% of GDM-related medical costs are covered by government programs, primarily Medicaid [26]. Furthermore, expanded coverage of emergency Medicaid for prenatal care has been associated with a significant increase in the use of antidiabetic medications during pregnancy among Latina patients with GDM or preexisting diabetes [41]. In summary, the relationship between SES and GDM, while requiring further examination, highlights the importance of addressing these complex interactions in maternal healthcare.

### Strengths and limitations

The strengths of this study include a large 15-year data set and, to our knowledge, the first analysis to assess Kansas specific GDM prevalence via rurality, and associated risk among demographic variables. This analysis included a cohort of all live births in Kansas from 2005 to 2019. This study has limitations, including the omission of factors such as parity, history of GDM, smoking, alcohol use, nutrition, and physical activity, which are recognized risk factors for GDM but were not the primary focus [4, 7, 42, 43]. Furthermore, the analysis did not account for variations in GDM screening and diagnosis criteria, making it challenging to determine accurate prevalence. In the US, different diagnostic criteria yield GDM diagnosis rates ranging from 5–20%, and changes in screening recommendations in 2014 may contribute to differences in prevalence over time or across jurisdictions [3, 26]. Additionally, there is the potential for underreporting of GDM, with birth certificate and hospital discharge data showing sensitivity ranging from 46–83% and often failing to adequately capture diabetes-complicated births [44]. This underreporting of health conditions, particularly in birth certificate data, could lead to an underestimation of both preexisting and gestational diabetes prevalence [3, 19]. Furthermore, the independence assumption of logistic regression could not be ensured given that the dataset provides unique identifiers per birth but not per mother, thus preventing the identification of multiple births of the same person. In populations where multiple births to the same individual are common, the above analysis may not effectively address the clustering arising from multiple live births within the same birthing woman. This may lead to odds ratio estimates that do not fully reflect the true associations about the risk factors associated with GDM and a higher Type 1 error due to underestimating the standard error.

### Public health implication

The rising prevalence of GDM and maternal obesity over 15 years highlight the need for increased alternatives in GDM screening and management, especially among at-risk demographics. This is further exacerbated by persistent rural-urban disparities demonstrated among GDM rates, emphasizing the necessity for targeted public health initiative such as policy changes, promotion of mobile health units, and attention to care in rural areas of Kansas. This study’s initial insight into the possible link between SES and GDM risk emphasizes the importance of addressing education, employment, and income to help mitigate the probability of GDM development. Lastly, the increased prevalence of maternal obesity demonstrates the need for promotion of lifestyle modifications, nutrition education, and physical activity to prevent both maternal obesity and GDM, ultimately improving maternal health outcomes.

## Conclusion

In summary, the overall prevalence of GDM and maternal obesity has risen over the years, displaying significant variations between rural and urban areas among Kansas women from 2005 to 2019. Furthermore, this research examined the GDM risk factors, accentuating the persistent racial/ethnic disparities and providing insight into socioeconomic status. We emphasize the call to action for ongoing endeavors and development of culturally responsive interventions for these particular subgroups to enhance maternal health and the wellbeing of their infants.

## Data Availability

The data that support the findings of this study are available from the Kansas Department of Health and Environment, but restrictions apply to the availability of these data, which were used under license for the current study, and so are not publicly available. Data are however available with permission of the Kansas Department of Health and Environment.

## Abbreviations

GDM: Gestational Diabetes Mellitus
BMI: Body Mass Index
KDHE: Kansas Department of Health and Environment
SES: Socioeconomic Status
